# Targeting lymphatics in inflammatory bowel disease

**DOI:** 10.18632/oncotarget.6026

**Published:** 2015-10-07

**Authors:** Silvia D'Aiessio, Carlotta Tacconi, Silvio Danese

**Affiliations:** Humanitas Clinical and Research Center, IBD Center and Humanitas University, Rozzano, Italy

**Keywords:** IBD, VEGFC, lymphatic function, macro phages

The inflammatory bowel diseases (IBDs), consisting of ulcerative colitis (UC) and Crohn's disease (CD), are characterized by chronic inflammation of the gastrointestinal tract in genetically susceptible individuals exposed to environmental risk factors. Although the precise pathophysiology of IBD is unknown, alterations in the intestinal lymphatic network such as lymphangiogenesis and lymphatic vessel (LV) dysfunction, are well-established features of human and experimental IBD (1). Such lymphangiogenic expansion might enhance classic intestinal lymphatic transport, eliminating excess accumulations of fluids, inflammatory cells and mediators, and could therefore be interpreted as an “adaptive” response to acute and chronic inflammatory processes. However, whether these new LVs are functional, dysregulated or immature is currently an area under investigation.

Together with LV dysfunction, macrophages (MΦs) have also a fundamental contribution to IBD pathogenesis. They have been found to play opposing roles in mouse models of intestinal inflammation. In fact, while classically activated M1 MΦs contribute to the exacerbation of the inflammation in mouse models of colitis (2), alternatively activated M2 MΦs contribute to the resolution of the disease (3). Thus, the factors that modulate MΦ polarization could affect the severity of human and experimental colitis. These observations suggest that resolution of chronic inflammation may require restoration of proper lymphatic function and proper MΦ activation, thus maintaining normal flow balance and helping the removal of inflammatory cells, mediators, and bacterial antigens away from inflamed sites.

Lymphangiogenesis is mediated by binding of the lymphatic vascular endothelial selective growth factors VEGF-C and VEGF-D to VEGFR3. Anti-lymphatic treatment with anti-VEGFR3 antibodies in an animal model of IBD has been shown to aggravate inflammation and submucosal edema, increase leukocyte infiltration, and to cause tortuous LVs (4). Besides, VEGF-C is also chemotactic for MΦs during pathological conditions, with its receptor VEGFR3 expressed by a substantial fraction of peripheral blood monocytes and activated tissue MΦs (5). Overall, these studies have established a direct role for VEGF-C/VEGFR3 signaling in both inflammation-induced lymphangiogenesis and immune response and suggest that therapies aimed at promoting lymphatic function, e.g., with prolymphangiogenic factors, such as VEGF-C, may provide a novel strategy for the treatment of inflammatory conditions, including IBD.

Recently, we examined the effect of stimulating lymphatic function and adaptive immune response via VEGF-C/VEGFR3 signaling on the severity of intestinal inflammation, on lymphatic drainage, as well as on bacterial antigen clearance and MΦ activation during inflammatory conditions. Furthermore, we evaluated the mechanism through which this pathway acts in experimental disease progression. We provided evidence, for the first time, that the specific promotion of LV function limits experimental chronic intestinal inflammation in mice; this is mediated by a unique MΦ polarization and activation, accompanied by modification of the tissue cytokine milieu (6). We reported that systemic inhibition of VEGFR3 blocked lymphangiogenesis, reducing both area density and LV dimension and growth, while significantly increasing inflammatory edema formation and impeding disease resolution. In contrast, lymphatic drainage was enhanced by systemic delivery of VEGF-C, which in turn significantly induced LV density and proliferation, improving intestinal inflammation.

Interestingly, the enhanced lymphatic drainage by VEGF-C was observed in combination with increased inflammatory cell mobilization and bacterial antigen clearance, all LV functions that we found to be inhibited by VEGFR3 blockade. VEGF-C–dependent antigen clearance was a MΦ-specific effect, thus demonstrating a potential role for VEGFR3 signaling in immunity by mediating antigen-presenting cell (APC) trafficking through MΦ recruitment.

The most intriguing and novel finding in this study is that the protection we observed in VEGF-C–treated mice during disease progression was not only a consequence of increased lymphangiogenesis and enhanced lymphatic flow and function, but was also a result of a previously unknown direct VEGF-C–induced MΦ activation through STAT6 signaling. This is intriguing, because some reports suggest that there may be a dysregulation of STAT6 signaling in the ungoverned immune response associated with colitis, and this transcription factor plays a regulatory role in the pathogenesis of IBD (7).

**Figure 1:**
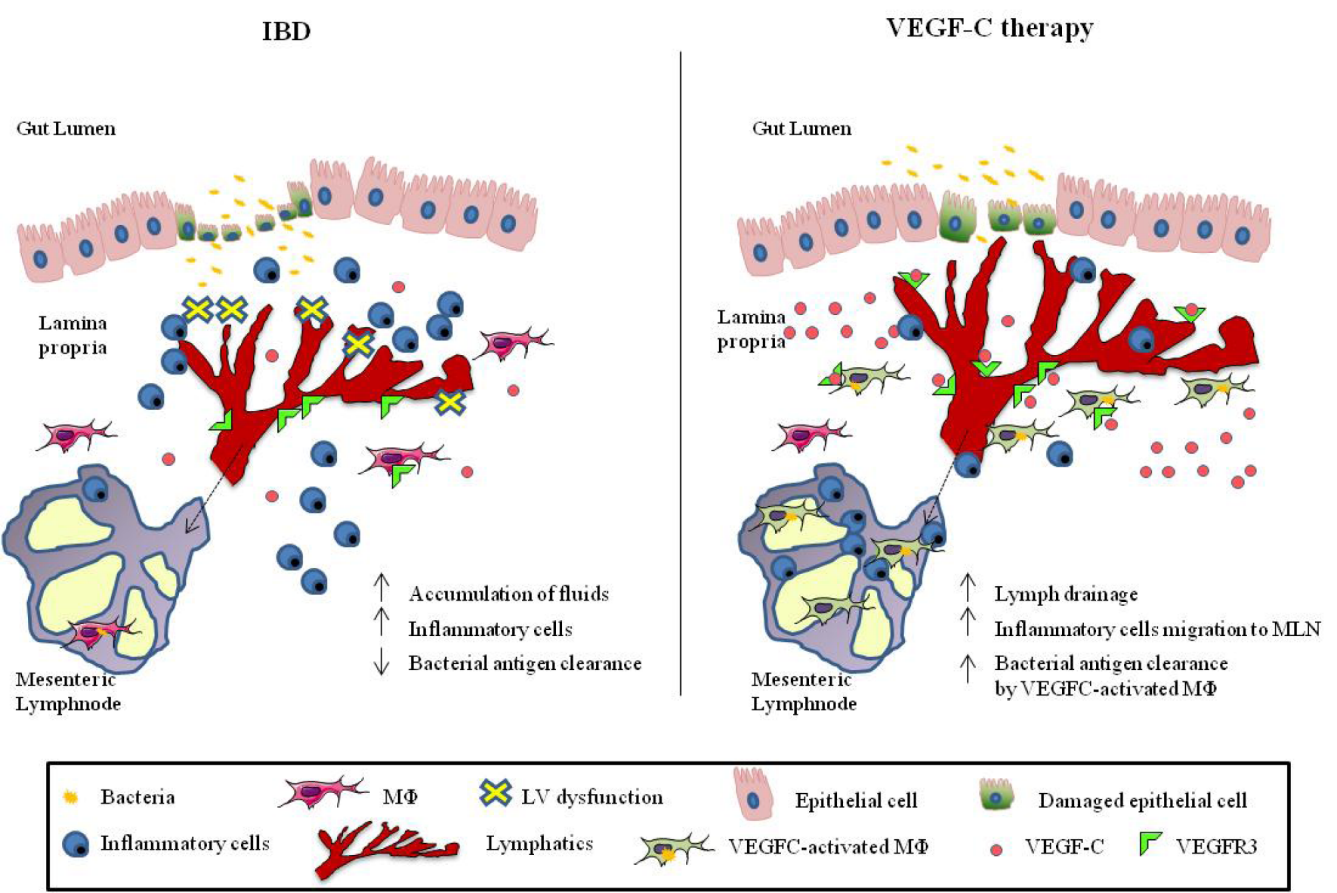
Potential use of lymphangiogenic factors (VEGF-C) as a novel therapeutic approach for the treatment of IBD IBD tissues show lymphatic dysfunction, which leads to accumulation of fluids, inflammatory cells and reduced bacterial antigen clearance (left panel). VEGF-C treatment induces lymph drainage and inflammatory cell migration towards draining lymphnodes (MLN), along with activation of macrophages (MΦ) and bacterial antigen clearance (right panel), in a STAT6-dependent manner. Dotted black arrow: lymph drainage.

In conclusion, our study provides the first proof of concept to our knowledge that it may be possible to treat chronic gastrointestinal inflammatory disorders by stimulating LV functions to promote bacterial antigen clearance, drainage of fluids and inflammatory cells, together with adaptive immunity, effects achievable through modulation of the VEGF-C/VEGFR3 pathway, as shown schematically in Figure 1. These findings are important because support the potential use of lymphangiogenic growth factors as a novel therapeutic approach for the treatment of IBD and other chronic inflammatory diseases.
